# Biases in detection of apparent “weekend effect” on outcome with administrative coding data: population based study of stroke

**DOI:** 10.1136/bmj.i2648

**Published:** 2016-05-16

**Authors:** Linxin Li, Peter M Rothwell

**Affiliations:** Stroke Prevention Research Unit, Nuffield Department of Clinical Neurosciences, John Radcliffe Hospital, University of Oxford, OX3 9DU, UK

## Abstract

**Objectives** To determine the accuracy of coding of admissions for stroke on weekdays versus weekends and any impact on apparent outcome.

**Design** Prospective population based stroke incidence study and a scoping review of previous studies of weekend effects in stroke.

**Setting** Primary and secondary care of all individuals registered with nine general practices in Oxfordshire, United Kingdom (OXVASC, the Oxford Vascular Study).

**Participants** All patients with clinically confirmed acute stroke in OXVASC identified with multiple overlapping methods of ascertainment in 2002-14 versus all acute stroke admissions identified by hospital diagnostic and mortality coding alone during the same period.

**Main outcomes measures** Accuracy of administrative coding data for all patients with confirmed stroke admitted to hospital in OXVASC. Difference between rates of “false positive” or “false negative” coding for weekday and weekend admissions. Impact of inaccurate coding on apparent case fatality at 30 days in weekday versus weekend admissions. Weekend effects on outcomes in patients with confirmed stroke admitted to hospital in OXVASC and impacts of other potential biases compared with those in the scoping review.

**Results** Among 92 728 study population, 2373 episodes of acute stroke were ascertained in OXVASC, of which 826 (34.8%) mainly minor events were managed without hospital admission, 60 (2.5%) occurred out of the area or abroad, and 195 (8.2%) occurred in hospital during an admission for a different reason. Of 1292 local hospital admissions for acute stroke, 973 (75.3%) were correctly identified by administrative coding. There was no bias in distribution of weekend versus weekday admission of the 319 strokes missed by coding. Of 1693 admissions for stroke identified by coding, 1055 (62.3%) were confirmed to be acute strokes after case adjudication. Among the 638 false positive coded cases, patients were more likely to be admitted on weekdays than at weekends (536 (41.0%) *v* 102 (26.5%); P<0.001), partly because of weekday elective admissions after previous stroke being miscoded as new stroke episodes (267 (49.8%) *v* 26 (25.5%); P<0.001). The 30 day case fatality after these elective admissions was lower than after confirmed acute stroke admissions (11 (3.8%) *v* 233 (22.1%); P<0.001). Consequently, relative 30 day case fatality for weekend versus weekday admissions differed (P<0.001) between correctly coded acute stroke admissions and false positive coding cases. Results were consistent when only the 1327 emergency cases identified by “admission method” from coding were included, with more false positive cases with low case fatality (35 (14.7%)) being included for weekday versus weekend admissions (190 (19.5%) *v* 48 (13.7%), P<0.02). Among all acute stroke admissions in OXVASC, there was no imbalance in baseline stroke severity for weekends versus weekdays and no difference in case fatality at 30 days (adjusted odds ratio 0.85, 95% confidence interval 0.63 to 1.15; P=0.30) or any adverse “weekend effect” on modified Rankin score at 30 days (0.78, 0.61 to 0.99; P=0.04) or one year (0.76, 0.59 to 0.98; P=0.03) among incident strokes.

**Conclusion** Retrospective studies of UK administrative hospital coding data to determine “weekend effects” on outcome in acute medical conditions, such as stroke, can be undermined by inaccurate coding, which can introduce biases that cannot be reliably dealt with by adjustment for case mix.

## Introduction

Many studies across different diseases have assessed whether there is a higher death rate after admission to hospital during the weekend (the “weekend effect”), with conflicting results.^1^ ^2^ ^3^ ^4^ ^5^ ^6^ The existence of a weekend effect would have important implications for patients, clinicians, and policy makers, and media coverage might deter patients from seeking medical attention at the weekend. Most previous studies that have attempted to study the effect, however, have used only routinely collected administrative data from hospital diagnostic coding,^1^ ^2^ ^3^ ^4^ ^5^ and few were able to quantify differences in the severity of the baseline event between weekday and weekend admissions.^6^ ^7^

Acute stroke has been most often used to study the weekend effect perhaps because of its high early mortality and the proved impact of quality and intensity of care on outcome.^8^ Reports of a weekend effect on stroke outcome have also been conflicting, but recent data on case fatality in acute hospital admissions in the United Kingdom, including stroke, have been used to inform proposed policy changes in provision of weekend service.^3^ This work, and most previous studies in the UK, however, was based on routinely collected administrative coding data, which can be inaccurate.^9^ ^10^ ^11^ ^12^ ^13^ ^14^ Indeed, the highest rates of inaccurate coding are found for acute medical admissions,^13^ ^14^ partly because patients are often elderly and have multiple active problems. This group of patients also has high short term mortality for the same reasons and will often “drive” analyses of weekend effects. Such analyses would therefore be expected to be particularly susceptible to biases secondary to coding errors.

No study has determined whether there are any differences in accuracy of coding for weekday and weekend admissions for acute stroke or any other condition. If any such differential accuracy of coding was also related to case fatality then it could explain apparent weekend effects. In particular, “false positive” coding, such as elective admissions after stroke or non-stroke admissions being coded as acute stroke admissions, could be particularly problematic but difficult to detect without prospective validation of all events. We therefore carried out a prospective population based study of the accuracy of hospital coding in acute stroke admissions on weekdays and at weekends and the potential impact of any inaccuracies on apparent outcome. We also assessed patient behaviour (that is, seeking medical attention) and other potential biases that might lead to apparent weekend effect and carried out a scoping review of previous studies of weekend effects in stroke.

## Methods

The Oxford Vascular Study (OXVASC) is an ongoing population based study of the incidence and outcome of all acute vascular events.^15^ The study population comprises all 92 728 individuals, irrespective of age, registered with about 100 general practitioners (GPs) in nine general practices in Oxfordshire, UK. Multiple overlapping methods of “hot” and “cold” pursuit were used to achieve near complete ascertainment of all individuals with transient ischaemic attack (TIA) or stroke.^15^ ^16^ These include daily rapid access “TIA and stroke clinic” to which participating GPs and the local emergency department refer individuals with suspected TIA or minor stroke; daily searches of admissions to the medical, stroke, neurology, and other relevant wards, also including screening all patients undergoing elective or emergency coronary, carotid, or peripheral vascular investigations or interventions; daily searches of the attendance register of the local emergency department; daily searches of records of deaths in hospital through the bereavement office; monthly searches of all death certificates and coroner’s reports for deaths outside hospital; monthly searches of GP diagnostic coding and hospital discharge codes (ICD-10 (international classification of diseases, 10th revision) codes I60-I68, G45-G46, H34); and monthly searches of all brain and vascular imaging referrals. Stroke was defined as rapid onset symptoms and/or signs of focal, and at times global, loss of cerebral function, with symptoms lasting more than 24 hours or leading to death, with no apparent cause other than of vascular origin.^15^

Study physicians saw patients as soon as possible after the initial presentation to determine dates and times of symptom onset, when medical attention was sought, and when patients were admitted to hospital or assessed in an outpatient clinic. Baseline demographic data, vascular risk factors, and other comorbidities were collected from face-to-face interview and cross referenced with primary care records. Detailed clinical history was recorded in all patients and assessments were made for stroke severity with the National Institute of Health Stroke Scale (NIHSS). Major stroke was defined as a score of ≥5. Patients routinely had brain imaging (computed tomography (CT) or magnetic resonance imaging (MRI)), vascular imaging (carotid Doppler or CT angiography/MRI angiography or digital subtraction angiography), 12 lead electrocardiography and standard blood tests. Echocardiography, 24 hour electrocardiography (HOLTER), and five day ambulatory electrocardiographic monitoring were done when clinically indicated. If a patient died before assessment, we obtained an eyewitness account of the clinical event and reviewed any relevant records. A senior neurologist (PMR) reviewed all cases for final adjudication, and reasons for exclusion were recorded.

A study nurse or physician followed up all patients face-to-face at 1, 6, 12, 60, and 120 months to determine recurrent strokes and functional status (modified Rankin scale, mRS). Patients who had moved out of the study area were followed up by telephone. All patients were flagged for Office for National Statistics mortality data, and all deaths during follow-up were recorded with causes. All patients with recurrent strokes who presented to medical attention would also be identified by the ongoing daily case ascertainment. If a recurrent stroke was suspected, a study physician re-assessed and investigated the patient.

### Statistical analyses

For all strokes ascertained in OXVASC in 2002-14 we compared the completeness of case ascertainment when only hospital discharge coding data were used versus when all sources were used and all events were adjudicated. As part of the “cold pursuit” methods in OXVASC, clinical adjudication was performed for all potential cases identified from hospital discharge coding with ICD-10 codes I60-I68, G45-G46, and H34 at any diagnostic position, however, for the purpose of the current study, and particularly to avoid overestimation of false positive cases, we considered only cases identified by codes I60-I68 as the primary diagnosis for coding identified cases. We also reported the number of false negative cases that could be identified by coding if other diagnostic positions were used.

The weekend was defined as the period from midnight on Friday to midnight on Sunday. All other times were defined as weekdays.^1^ We compared all the admissions for stroke in the study population identified by coding only with all adjudicated OXVASC strokes ascertained in the same period (2002-14) and compared the differences in accuracy (false negative and false positive cases) and reasons for inaccurate coding between weekday and weekend admissions using χ^2^ test. We calculated the 30 day case fatality (%) using coding data only and compared the impact of different coding selection criteria on 30 day case fatality between weekday and weekend admissions. Sensitivity analyses confined to ICD-10 codes I60-I64 or I60, I61, I62.9, I63, and I64 were performed. We also further applied the additional information on admission method (elective *v* emergency) from coding and included only coding identified emergency admissions for sensitivity analyses.

For all incident strokes in OXVASC, we compared day of the week of onset versus day of the week when medical attention was sought, stratified by severity of stroke (minor *v* major) using Poisson regression analyses. For all OXVASC patients with stroke who were admitted to hospital, we used χ^2^ test to compare the case fatality at seven days and 30 days between weekend and weekday admissions. Logistic regression analysis was used to adjust for age, sex, baseline disability, and event severity (NIHSS at baseline). Sensitivity analyses including only incident strokes were also performed. Using data on functional status (modified Rankin score) at follow-up for all patients with incident stroke, we also used ordinal regression analysis to compare the status of patients admitted at the weekend and weekdays adjusted for age, sex, baseline disability, and event severity (NIHSS).

All analyses were performed with SPSS version 20.

### Search strategy and data extraction for scoping review

We searched PubMed for articles on weekend effect in hospital admissions for stroke published from January 2000 to February 2016, using the search terms “stroke”, “cerebrovascular disease”, “weekend effect”, “weekend”, “off-hour”, and “outcome”. We also reviewed the references of all identified studies. We included any study that reported the short term (in hospital; seven day or 30 day) outcome (death or functional outcome) of patients admitted with stroke by day of the week (that is, weekdays versus weekend; workings hour versus out of hours). Studies that reported only the service differences by day of the week or studies that were restricted to patients with stroke undergoing thrombolysis or endovascular treatment were excluded. There was no language restriction. For each study, we extracted details on general study characteristics (study name, study period, year of publication, and study type), information on the study population (country, number of patients included, and patient demographics), and details on the outcome measure (death or functional outcome). Moreover, we documented the following factors that could potentially bias the observed weekend effect: source of the study dataset, descriptions of the ICD codes used if data source included administrative coding data, and if information on admission method was used for the administrative dataset to exclude elective/scheduled admissions; how readmission after the first stroke admission was dealt with; whether stroke severity was assessed; and whether comorbidities of the patients were measured.

Among all eligible studies, we used χ^2^ test to investigate the associations of reporting a weekend effect and different study characteristics (that is, study population, study period, data source, whether event severity was assessed and whether comorbidity was adjusted for). Given the potential impact of unmeasured case mix factors at baseline (such as imbalance of age, baseline disability, and severity of stroke in weekday versus weekend admissions) on the weekend effect, we carried out a pooled analyses including only clinical registries with detailed measurement of case severity using Mantel-Haenzel-Peto method (random effect). We included all reported adjusted odds ratios for short term outcome (death) in weekend versus weekday comparisons when possible. For studies that did not report any adjusted estimate, we used unadjusted odds ratios.

### Patient involvement

No patients were involved in setting the research question or the outcome measures, nor were they involved in recruitment or the design and implementation of the study. There are no plans to involve patients in the dissemination of results.

## Results

Among the 92 728 patients in the study population, 2373 strokes were ascertained in OXVASC for 2002-14, of which 826 (34.8%) were managed in clinic or at home and 60 (2.5%) happened out of the area or abroad and would therefore not be identified if only coding data were used. A further 195 strokes (8.2%) happened during admissions for another reason and were coded with the date of the admission for the initial diagnosis. Among the 1292 remaining patients who were admitted to the local hospital for acute stroke, only 973 (75.3%) were identified by coding. There was no bias in distribution of weekend and weekday admission for the 319 strokes missed by coding—that is, false negatives (227 (24.1%) on weekday *v* 92 (26.2%) at weekend; P=0.44). Table 1 shows the reasons for strokes not being identified by coding. Results were similar in the analysis confined to incident strokes ascertained in OXVASC (table 1).

**Table 1 tbl1:** Strokes ascertained in Oxford Vascular Study that were not identified by hospital diagnostic coding. Figures are numbers (percentage) of patients

	All strokes (n=2373)				All incident strokes (n=1849)		
	Weekday	Weekend	Total		Weekday	Weekend	Total
**All strokes**							
No of patients	1745	628	2373		1349	500	1849
Patients not admitted	619 (35.5)	207 (33.0)	826 (34.8)		471 (34.9)	157 (31.4)	628 (34.0)
Strokes that happened during admission for other events	153 (8.8)	42 (6.7)	195 (8.2)		93 (6.9)	30 (6.0)	123 (6.7)
Strokes that happened out of area or abroad	38 (2.2)	22 (3.5)	60 (2.5)		37 (2.7)	23 (4.6)	60 (3.3)
Patients admitted to hospital	935 (53.6)	357 (56.8)	1292 (54.4)		748 (55.4)	290 (58.0)	1038 (56.1)
**Strokes in patients admitted to hospital**							
No of patients	941	351	1292		748	290	1038
Identified by coding from primary diagnosis	714 (75.9)	259 (73.8)	973 (75.3)		565 (75.5)	217 (74.8)	782 (75.3)
Missed by coding	227 (24.1)	92 (26.2)	319 (24.7)		183 (24.5)	73 (25.2)	256 (24.7)
Identified by coding but not from primary diagnosis	3 (0.3)	3 (0.8)	6 (0.5)		2 (0.3)	3 (1.0)	5 (0.5)
Missed by coding‡	154 (16.4)	59 (16.8)	213 (16.5)		120 (16.0)	44 (15.2)	164 (15.8)
Inaccurately coded as transient ischaemic attack	44 (4.6)	21 (5.9)	65 (5.0)		35 (4.7)	18 (6.2)	53 (5.1)
Inaccurately coded with other diagnosis§	23 (2.4)	8 (2.3)	31 (2.4)		23 (3.1)	7 (2.4)	30 (2.9)
Coding as admitted but was admitted only for investigation or early recurrence	3 (0.3)	1 (0.3)	4 (0.3)		3 (0.4)	1 (0.3)	4 (0.4)

*Based on day of week for onset.

†Based on day of week of admission.

‡True acute stroke admissions to hospital that were not matched to any administration data records obtained from same hospital.

§Such as subdural haemorrhage, occlusion of cerebral artery, cerebral aneurysm without rupture, myocardial infarction, dementia, collapse, brain injury, dysphagia.

There were 1693 admissions for stroke identified by hospital discharge coding, among which 290 (17.1%) episodes were recurrent admissions for stroke after the first admission. After case adjudication, 1055 (62.3%) events were considered to be accurate, among which 53 (3.1%) were in patients who had already been managed by physicians in the emergency department on the previous day while the patient was waiting to be admitted to a ward. False positive diagnostic coding was more common in admissions on weekdays than at the weekend (536 (41.0%) *v* 102 (26.5%); P<0.001). Among the 638 false positive cases, the reasons for inaccurate coding also differed between weekdays and weekend admissions (table 2), with more admissions for investigation or procedure for previous stroke miscoded as new acute stroke episodes on weekdays (34.1% *v* 12.7%, P<0.001; table 2) and more inaccurate admission dates recorded for weekend admissions (25.5% *v* 8.6%; P<0.001; table 2). Sensitivity analyses confined to ICD-10 codes I60-I64 (table A in appendix 1) or I60, I61, I62.9, I63, and I64 (table B in appendix 1) showed consistent results.

**Table 2 tbl2:** Differences in 30 day case fatality, frequency, and reasons for inaccurate coding of stroke admissions during weekdays and at weekend

	Total	No (%) of deaths at 30 days	No (%) of admissions		P value
			Weekdays	Weekend	
**Correctly identified episodes by coding (true positive)**					
No of patients	1055	233 (22.1)	772	283	0.44
Incident stroke	787	167 (21.2)	571 (74.0)	216 (76.3)	—
Recurrent stroke	268	66 (24.6)	201 (26.0)	67 (23.7)	—
**Incorrectly identified episodes by coding (false positive)**					
No of patients	638	66 (10.3)*	536	102	<0.001
Cancelled admission	15	0 (0)	15 (2.8)	0 (0)	0.09
Elective admission	293	11 (3.8)	267 (49.8)	26 (25.5)	<0.001
Investigation or procedure only†	196	0 (0)	183 (34.1)	13 (12.7)	<0.001
Rehabilitation after stroke	63	4 (6.3)	55 (10.3)	8 (7.8)	0.45
Transferred from other hospital	34	7 (20.6)	29 (5.4)	5 (4.9)	0.83
Non-stroke diagnoses	226	29 (12.8)	183 (34.1)	43 (42.2)	0.12
Medical problem post stroke discharge	23	6 (26.1)	19 (3.5)	4 (3.9)	0.85
Subdural/extradural haemorrhage	55	10 (18.2)	47 (8.8)	8 (7.8)	0.76
Other diagnosis‡	148	13 (8.8)	117 (21.8)	31 (30.4)	0.06
Admission date wrong	72	23 (31.9)	46 (8.6)	26 (25.5)	<0.001
Inpatient event after elective admission§	9	3 (33.3)	7 (1.3)	2 (2.0)	—
Inpatient event after emergency admission for other disease¶	39	17 (43.6)	24 (4.5)	15 (14.7)	—
Admission date wrong**	24	3 (12.5)	15 (2.8)	9 (8.8)	
General practitioner information wrong	20	0 (0)	16 (3.0)	4 (3.9)	0.62
Unknown	12	3 (25.0)	9 (1.7)	3 (2.9)	0.39

*P<0.001 for heterogeneity among groups.

†Including carotid endarterectomy, cerebral angiography, endovascular treatment for aneurysm, neurosurgery, brain imaging.

‡Including transient ischaemic attack, amaurosis fugax, trauma only, dementia, brain tumour, chronic small vessel disease, vasculitis, seizure, headache.

§For example, patient admitted for elective cardiac surgery on “admission date” but had stroke as inpatient on ward six days later.

¶For example, patient admitted during weekend for pneumonia and subsequently had acute stroke on Tuesday during same admission.

**Admission date for acute stroke from hospital coding data was not date that patient was actually admitted.

For all episodes identified by coding, there were 299 deaths at 30 days after hospital admission. Table 2 shows the differences in mortality stratified by coding accuracy. The 30 day case fatality differed significantly between accurate and false positive cases, with significantly lower mortality for the false positive cases (66 (10.3%) *v* 233 (22.1%); P<0.001; table 2), particularly for the miscoded elective admissions (11 (3.8%), table 2). As a result, weekday admission mortality changed with different case selection criteria, with the 30 day case fatality increased from 17.5% when all episodes were included to 22.9% when only stroke admissions that were correctly coded were included (P=0.001, fig 1). On the contrary, the case fatality at 30 days did not differ significantly for weekend admissions based on the selection criteria (P=0.59, fig 1). Moreover, when there were multiple admissions for a single patient, changing the selection criteria from the last admission to the first admission reversed the apparent weekend effect (fig 1).

**Figure f1:**
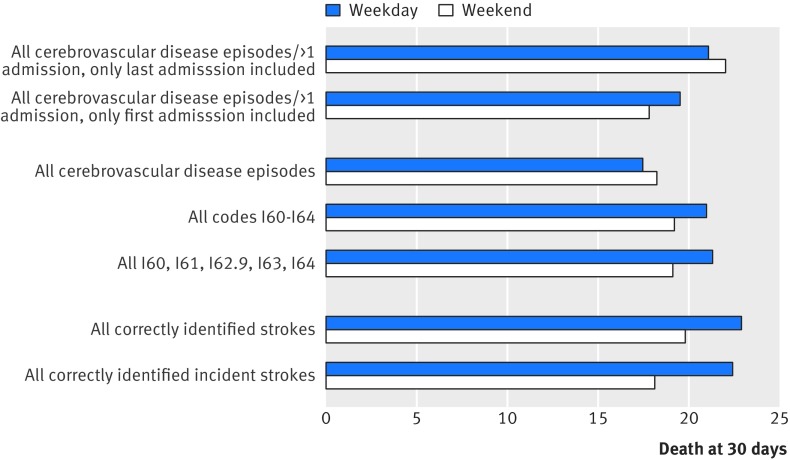
**Fig 1** Case fatality at 30 days in weekday *v* weekend admissions stratified by different coding selection criteria

In addition to the use of standard ICD-10 codes to identify stroke admissions, method of admission (that is, elective *v* emergency), when available, could also be used to reduce the false positive rate. Among the 1661 (98.1%) cases with admission method adjudicated by OXVASC, the overall accuracy for coding to record the admission method was good (1564, 94.2%). Among the 1327 emergency admissions for stroke identified by coding (79.9% of all admissions), however, 238 (17.9%) were false positive cases with low expected case fatality, including 40 elective admissions that were miscoded as emergency cases and 198 non-stroke acute admissions (admissions for transient ischaemic attack, headache with negative imaging, amaurosis fugax, or chronic small vessel disease; table C in appendix 1). As expected, the 30 day case fatality in these 238 cases was significantly lower than in the true cases (35/238 (14.7%) *v* 231/1042 (22.2%); P=0.01; table C in appendix 1). More importantly, there were significantly more cases with low expected case fatality in weekday than in weekend admissions (190/976 (19.5%) *v* 48/351 (13.7%); P<0.02; table C in appendix 1). Similarly, although the number was small (47, 3.5%; table C in appendix 1) for false positive cases with high expected case fatality (that is, acute stroke that happened during an inpatient admission for other acute diseases), there were more cases of high expected case fatality being miscoded as acute stroke at weekends (22 (6.3%) at weekend *v* 25 (2.6%) on weekday; P=0.001; table C in appendix 1).

Another potential bias with the use of administrative coding data alone would be unbalanced event severity for weekday versus weekend admissions because of differences in patient behaviour. For all incident strokes ascertained by OXVASC, we recorded the day of the week of onset and the day when medical attention was sought. Day of onset was uniform (P=0.70; fig 2), but patients were less likely to present for medical attention at the weekend than on weekdays (P<0.001; fig 3). When the analysis was stratified by severity of stroke, patients with major strokes were equally likely to present for medical attention on weekdays and at the weekend (P=0.51; fig 3), while those with minor strokes were less likely to present at the weekends (P<0.001; fig 3). Therefore, among all strokes in patients who presented for medical attention, there were proportionally more major strokes at the weekend than on weekdays (165 (42.9%) *v* 471 (35.6%), odds ratio 1.36, 95% confidence interval 1.08 to 1.71; P=0.01). While patients with major stroke tended to use emergency services (directly presenting to emergency department/NHS direct/ambulance) equally when presenting during the weekend and weekdays (125/75.8% *v* 332/70.5%; P=0.20), those with minor events were more likely to use emergency services when they presented at the weekend (119 (54.1%) *v* 216 (25.4%); P<0.001) and were thus more likely to be admitted to hospital (fig 3). Therefore among patients admitted to hospital, the proportion with major stroke did not differ between weekday and weekend admissions (392 (52.4%) *v* 149 (51.4%); P=0.77). There was also no imbalance in the distribution of NIHSS at presentation on weekday versus weekend admissions (fig 4 [Fig f4]and fig B in appendix 2).

**Figure f2:**
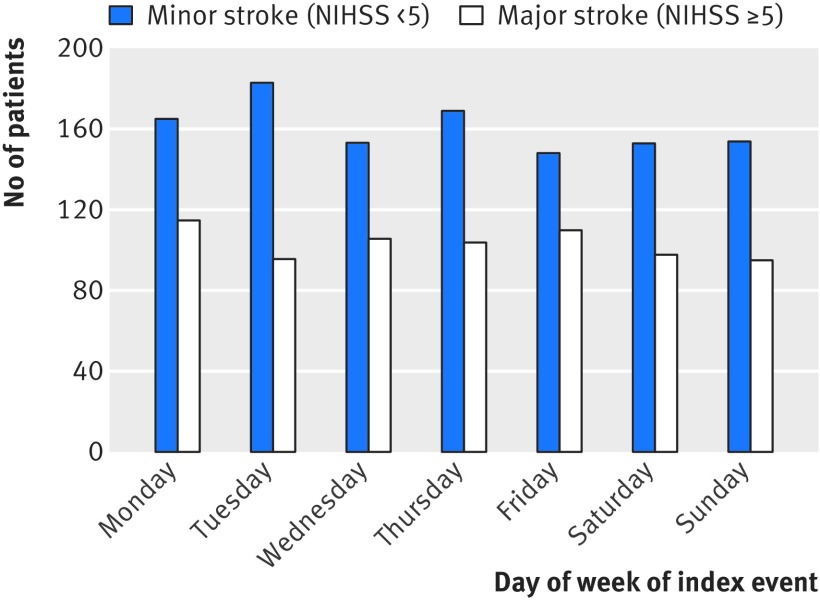
**Fig 2** Number of patients with stroke by day of week of onset stratified by stroke severity (NIHSS score)

**Figure f3:**
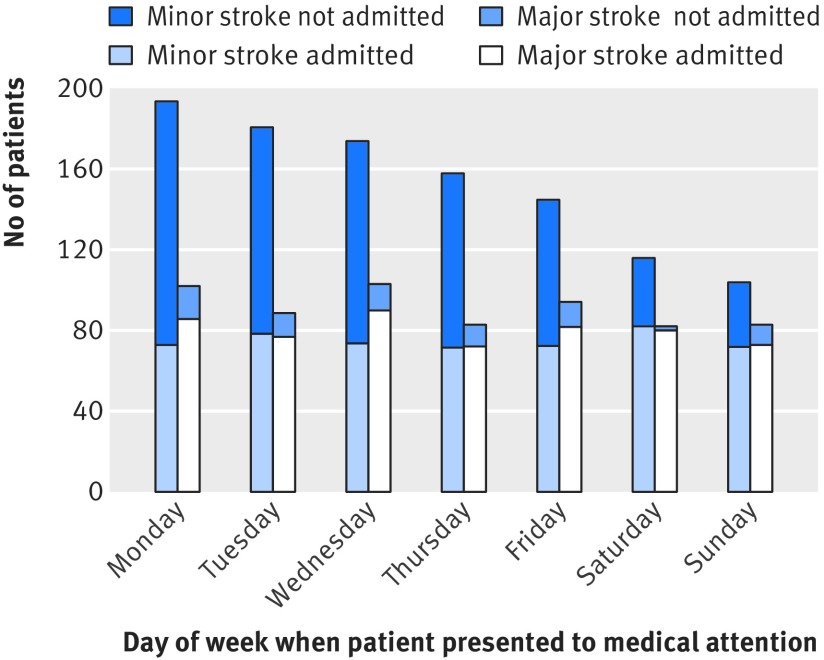
**Fig 3** Number of patients with stroke (admitted *v* not admitted) by day of week of presentation for medical attention stratified by stroke severity (NIHSS score)

**Figure f4:**
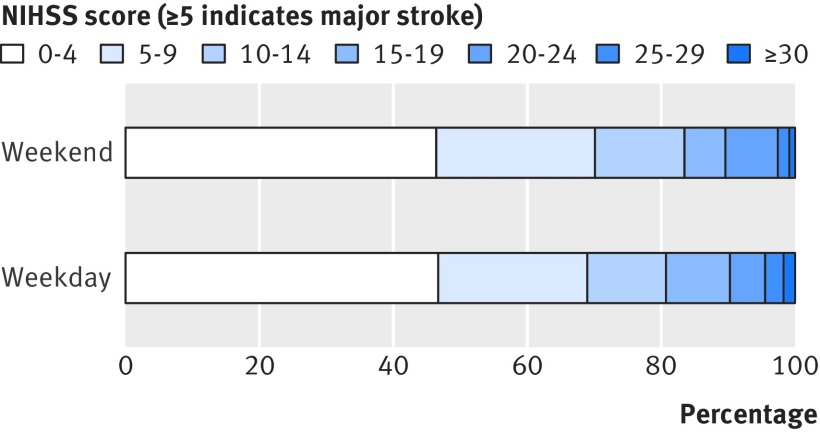
**Fig 4** Distribution of stroke severity (NIHSS score) in patients admitted during weekend *v* weekdays in OXVASC (odds ratio 0.97, 95% confidence interval 0.77 to 1.23; P=0.82)

Among all stroke admissions ascertained in OXVASC, we found no difference in case fatality for weekend and weekday admissions at seven days (odds ratio 0.87, 95% confidence interval 0.62 to 1.21; P=0.40; table 3) or at 30 days (0.90, 0.68 to 1.19; P=0.46; table 3). Results were similar after adjustment for age, sex, and severity of event (table 3). Analysis that excluded deaths in the emergency department and strokes in inpatients and analysis that included only incident strokes showed similar results (table 3). Moreover, we did not find any adverse weekend effect on the modified Rankin scale at one month (adjusted odds ratio 0.78, 0.61 to 0.99; P=0.04; fig 5 and fig A in appendix 2) or one year (0.76, 0.59 to 0.98; P=0.03) among incident strokes.

**Table 3 tbl3:** Outcomes of weekend versus weekday admissions for strokes ascertained in Oxford Vascular Study

	Weekend	Weekday	Odds ratio (95% CI)				
			Unadjusted	P value		Adjusted	P value
**All patients with stroke admitted in hospital**							
No of patients	394	1098	—	—		—	—
Death at 7 days	51 (12.9)	161 (14.7)	0.87 (0.62 to 1.21)	0.40		0.82 (0.58 to 1.18)	0.29
Death at 30 days	86 (21.8)	260 (23.7)	0.90 (0.68 to 1.19)	0.46		0.85 (0.63 to 1.15)	0.30
**All patients with stroke admitted to hospital, excluding death at emergency department and inpatient events**							
No of patients	351	941	—	—		—	**—**
Death at 7 days	37 (10.5)	123 (13.1)	0.78 (0.53 to 1.16)	0.22		0.75 (0.50 to 1.13)	0.17
Death at 30 days	67 (19.1)	195 (20.7)	0.90 (0.66 to 1.23)	0.52		0.87 (0.62 to 1.22)	0.41
**All patients with incident stroke admitted in hospital**							
No of patients	320	853	—	—		—	—
Death at 7 days	41 (12.8)	130 (15.2)	0.82 (0.56 to 1.19)	0.29		0.78 (0.52 to 1.17)	0.23
Death at 30 days	65 (20.3)	206 (24.2)	0.80 (0.59 to 1.10)	0.17		0.75 (0.53 to 1.07)	0.11
**All patients with incident stroke admitted in hospital, excluding death at emergency department and inpatient events**							
No of patients	290	748	—	—		—	—
Death at 7 days	28 (9.7)	99 (13.2)	0.70 (0.45 to 1.09)	0.11		0.68 (0.42 to 1.08)	0.10
Death at 30 days	49 (16.9)	158 (21.1)	0.76 (0.53 to 1.08)	0.13		0.73 (0.50 to 1.08)	0.11

*Adjusted for age, sex, and severity of event.

**Figure f5:**
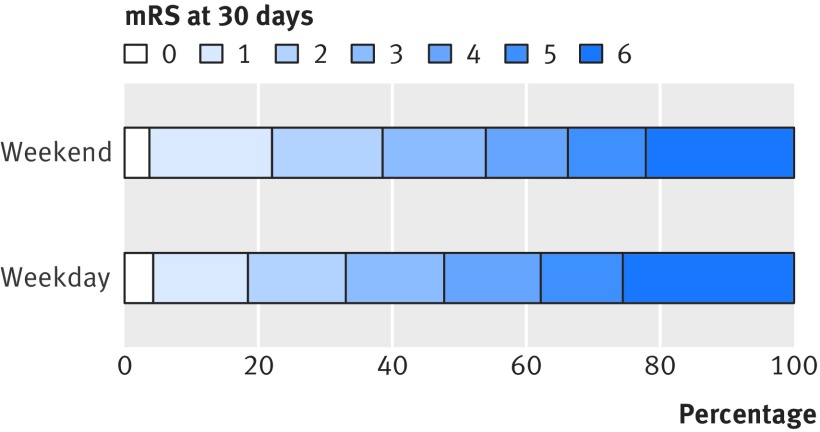
**Fig 5** Modified Rankin scale (mRS) at 30 days in patients admitted during weekend *v* weekdays in OXVASC

Among the 42 studies we identified in our scoping review that assessed if there was a weekend effect for patients admitted to hospital with acute stroke, 19 (45.2%) found a significant effect. Appendix 3 lists details of the studies. Most studies (n=39, 92.9%) were carried out after the introduction of stroke unit care. More than half (n=22, 52.4%) used administrative data only, particularly the studies published in the UK (six of seven studies). Although most studies adjusted for comorbidity (n=33, 78.6%), less than half (n=17, 40.4%) measured stroke severity. Compared with clinical registries, studies that used only administrative coding dataset were more likely to report a weekend effect (68% *v* 20%; P=0.002; table 4), while studies that adjusted for event severity were less likely to find a weekend effect than studies with no measurement of stroke severity (18% *v* 64%; P=0.003; table 4). In the 16 clinical registry studies that adjusted for event severity, only three (19%) reported a weekend effect. Moreover, in the pooled analysis, when only clinical registries with balanced baseline characteristics in weekend versus weekday admissions were included, we also found no weekend effect (pooled odds ratio 0.94, 95% confidence interval 0.80 to 1.10; P=0.45; table 5).

**Table 4 tbl4:** Frequency of reported weekend effect in previous studies stratified by factors that could potentially affect the results

	No of patients included*	No (%) of studies with significant weekend effect	P value†
Country:			
United Kingdom (n=7)	907 200	6 (86)	<0.05
United States (n=11)	1 121 703	3 (27)	
Other countries (n=24)	4 097 764	10 (42)	
Study period:			
Before 2002 (n=3)	54 106	1 (33)	0.91
After 2002 (n=30)	1 999 231	14 (47)	
Mixed (n=9)	4 073 330	4 (44)	
Data source:			
Administrative dataset (n=22):	5 747 120	15 (68)	0.002
Only emergency admissions included (n=4)	4 034 021	1 (25)	
Clinical registry (n=20)	379 547	4 (20)	
Event severity included:			
Yes (n=17)	190 527	3 (18)	0.003
No (n=25)	5 936 140	16 (64)	
Comorbidity included:			
Yes (n=33)	5 001 495	17 (52)	0.12
No (n=9)	1 125 172	2 (22)	

*One UK study using administrative dataset and one clinical registry from Spain did not report exact number for stroke patients included in analysis.

†For frequency difference between groups.

**Table 5 tbl5:** Pooled analyses of short term case fatality in weekend versus weekday admissions in clinical registries with measured stroke severity stratified by baseline patient characteristics including patient age, premorbid disability, and event severity

Study*	Country		Total No of patients	Odds ratio (95% CI)
**Studies with balanced baseline characteristics in weekday *v* weekend admissions**				
Albright^e9^	United States		2180	1.10 (0.74 to 1.63)
O’Brien^e20^	United States		929	0.87 (0.51 to 1.50)
Kim^e22^	Korea		1247	1.10 (0.64 to 1.86)
Martinez^e24^	Spain		674	1.08 (0.48 to 2.45)
Albright^e27^	United States		2085	0.84 (0.58 to 1.22)
Bejot^e29^	France		1582	1.00 (0.69 to 1.45)
OXVASC	United Kingdom		1492	0.85 (0.63 to 1.15)
**Studies with imbalanced or unknown baseline characteristics in weekday *v* weekend admissions**				
Hasegawa^e3^	Japan		1134	2.08 (1.25 to 3.45)
Jauss^e7^	Germany		37 396	1.00 (0.86 to 1.16)
Niewada^e23^	Poland		19 667	1.20 (1.10 to 1.32)
Nakajima^e40^		Japan	5625	1.15 (0.90 to1.46)
Fang^e15^	Canada		10 107	1.17 (1.00 to 1.38)§

*See appendix 3 for reference details.

†Pooled estimate 0.94 (0.80 to 1.10), P=0.45; heterogeneity I^2^=0%, P=0.92.

‡Pooled estimate 1.18 (1.00 to 1.39), P=0.45; heterogeneity I^2^=68%, P=0.02.

§Hazard ratio (95% CI).

## Discussion 

We identified several potential biases in detection of apparent “weekend effects” in outcome of stroke. Firstly, whereas patients with major stroke presented uniformly throughout the week, patients with minor stroke were less likely to present at the weekend. This could potentially affect weekday and weekend outcomes depending on local admission policies throughout the week. Secondly, about a third of patients with stroke were managed as outpatients and would not be identified by hospital coding. Thirdly, among all patients with stroke who were admitted to hospital, more than a third of the subsequent administrative diagnostic codes were inaccurate. Crucially, the rate of false positive coding differed between weekday and weekend admissions, with more elective weekday admissions with a low expected case fatality being miscoded as acute stroke. Although admission method, when available, could be used to reduce the false positive rate to some extent, there were still more cases with low expected case fatality being miscoded as acute stroke when only admissions coded as emergency were included. It is noteworthy, therefore, that in our scoping review of previous studies, the finding of a weekend effect was associated with studies that used only administrative coding. In contrast, no weekend effect on short term case fatality was apparent in prospective cohorts with clinical adjudication of stroke diagnosis that did not have an imbalance in severity of stroke on admission.

The finding of inaccuracy of hospital diagnostic coding is in line with findings in previous studies of acute admissions, with low sensitivity and specificity of coding data reported in cardiovascular disease,^17^ ^18^ aortic aneurysm,^9^ peripheral vascular disease,^10^ general surgery,^11^ and stroke.^12^ ^19^ ^20^ ^21^ ^22^ ^23^ The process and quality of coding varies from country to country. In the UK, hospital diagnostic coding is often done by non-clinical clerical staff and largely depends on their interpretation of medical notes and application of appropriate codes. The actual reason for the acute admission is not always clear in patients with multiple comorbidities, and elective admissions for investigation or management of previous disease are sometimes miscoded as admissions for acute events. Importantly in relation to studies of weekend effects, we found that the reasons for inaccurate coding differed between weekday and weekend admissions, with more weekday admissions with low risk of mortality being miscoded as acute stroke. Low risk admissions, such as elective procedures or investigations for previous TIA or stroke, were most likely to occur during weekdays, thereby reducing the overall case fatality of weekday admissions, resulting in an apparent weekend effect for stroke outcome if it is based on coding data alone.

One way to overcome the bias caused by the inclusion of elective cases according to coding data alone is to use the “admission method” information to exclude all elective admissions. Only six previous studies, however, reported that they applied this method (appendix 1). Although three out of the six studies still found a weekend effect, two reported only the results for all disease conditions combined but not for acute stroke specifically (appendix 1). Only one of the four studies that included only emergency admissions for stroke found a weekend effect. As expected, the admission method can also be recorded incorrectly. In our study, nearly 13% of true elective admissions were coded as emergency. Although miscoding elective as emergency by the admission method also happened more often for weekday admissions, as 80% of stroke admissions are emergency, miscoding elective as emergency on its own would have a rather small impact (2%) on the overall effect on outcome. In a condition for which there are more scheduled admissions for acute disease (for example, symptomatic aortic aneurysm), however, the effect could be bigger (table D in appendix 2). More importantly, we showed that even when the admission method was available to exclude all elective admissions, there were still more cases with low expected case fatality being included for weekday admissions.

Baseline difference in case mix is another potential bias in studies of the weekend effect. Although many studies attempt some adjustment for case mix, this is usually limited to the Charlson comorbidity index derived from coding data (appendix 3). In addition to limitations of coding in identifying comorbidities,^23^ ^24^ the most important case mix factors (stroke severity and premorbid functional status) are generally not available.^25^ Patients with minor stroke were less likely to present at weekends, in accordance with previous studies.^7^ ^26^ ^27^ As hospital admission rates might differ between weekdays and weekends,^5^ the difference of behaviour in patients with minor events could potentially affect the case severity in hospital admissions on weekdays versus weekends. In our study population, patients with minor stroke were more likely to use emergency services if they presented during the weekend and were therefore more likely to be admitted at the weekend. As a result, we did not find an overall excess of weekend admissions with major strokes, but the effect of this type of presentation bias could depend on local admission policies.

In the scoping review, when we included only clinical registries with balanced baseline characteristics in weekend versus weekday admissions, we did not find any weekend effect. The trend towards similar or even better outcome for weekend versus weekday admissions in our population is perhaps also due to the established 24/7 stroke service in our hospital, with better availability of imaging during the weekend, which also highlights the importance of implementation of quality improvement initiatives (such as dedicated specialised stroke networks) in attenuating inequalities in the management of patients with acute stroke.

### Strengths and limitations

The strength of our study was its population based design with multiple overlapping methods to achieve near complete ascertainment of all patients with acute stroke, using daily ascertainment in the acute phase as well as data extracted from medical notes, general practices, and national/hospital coding. Therefore we were able to study the potential reasons for miscoding and the effect of differences in patient behaviour and disease severity on the weekend effect.

There are several limitations of the analyses. Firstly, our study covered a period of more than 10 years, during which time coding accuracy might have improved.^28^ However, in our analyses stratified by study period, although coding accuracy did improve both for weekday and weekend admissions over time, the relative higher proportion of false positive cases in weekday versus weekend admissions remained consistent throughout the study periods (table E in appendix 1). Secondly, our hospital is a teaching hospital with good stroke service and thus one could argue that it might not be representative of all hospitals in the UK. In our analyses stratified by study periods, however, we found that the trend of a better outcome for weekend admissions was even more prominent during the early phase when the stroke service in our region was not fully established (table F in appendix 1). Moreover, other studies in the UK also reported poor accuracy of using administrative data alone in identifying acute stroke.^13^ ^29^ ^30^ Therefore it is likely that these coding related biases in detecting a weekend effect in acute stroke could also exist in other hospitals in the UK. Thirdly, though we used acute stroke as an example, similar biases are also likely to exist for other acute conditions if the weekend effect is studied with administrative data. Such data might be more satisfactory in identifying surgical procedures, cancer, or rare diseases, but in conditions for which patients are most likely to be admitted to hospital acutely, coding accuracy is known to be poor.^11^ ^14^ ^31^ Fourthly, as coding accuracy might differ between countries and healthcare systems, the coding related biases we reported using UK data might not be generalisable to other countries. Recent data from countries using different coding practices to the UK, however, also reported low sensitivity and positive predictive value with administrative data alone in identifying acute stroke.^19^ ^32^ ^33^ Fifthly, our study focused on the potential biases in using administrative data alone in detecting any weekend effect and these biases might not be generalisable in other clinical or research questions. Further improvement in coding accuracy will be important to support its use for research and managerial decision making, but even when coding is not accurate it can still be used to investigate some research questions. Finally, our scoping review of the previous literature was only a limited form of systematic review but was intended to be a guide to the likely generalisability of our study findings.

### Clinical implications and conclusions

Using acute stroke as an example, we showed potential biases that could lead to apparent weekend effects in a range of other acute conditions. Given the limitations of coding of acute medical admissions, at least in the UK, any conclusion based on administrative data alone should be interpreted with caution. Future studies examining the weekend effect should ideally be based on prospective studies of clinically confirmed cases or at least include some validation of coding data against a clinical ideal standard. If only administrative data are available, however, use of information on “admission method,” if available, application of a more stringent selection of ICD codes, and consideration of analyses limited to the first admission in any given study period could reduce bias caused by false positive cases. Our findings perhaps explain why previous studies of clinically confirmed stroke cases, and studies of coding data outside the UK, were less likely to find weekend effects.

**What is already known on this topic**

There is conflicting evidence as to whether mortality is higher during weekend compared with weekday admissions to hospital
Many of the previous studies assessing the weekend effect were based on administrative coding data alone, but the possibility that the accuracy of coding data might differ between weekend and weekday admissions has not previously been assessed

**What this study adds**

While unmeasured imbalances in baseline case mix, such as event severity and inpatient behaviour, are potential biases in reporting of the weekend effect, the main bias in coding based studies of stroke is inaccuracy of coding data (at least in the UK), particularly the inclusion as weekday admissions of false positive cases with low expected case fatality
Similar biases are likely to occur in studies of the weekend effect in acute admissions for other conditions for which administrative diagnostic coding is prone to inaccuracy

## Web Extra

Extra material supplied by the author

Appendix 2: Supplementary figures A-B

Appendix 3: Details of studies included in scoping review

## Notes

We thank all the staff working for the Oxford Vascular Study and all our collaborating general practices.

Contributors: LL acquired, analysed, and interpreted the data and drafted and revised the manuscript. PMR conceived, designed, and supervised the study, obtained funding, analysed and interpreted the data, and drafted and revised the manuscript. Both authors are guarantors and had full access to all of the data (including statistical reports and tables) in the study and take responsibility for the integrity of the data and the accuracy of the data analysis.

Ethical approval: The Oxford Vascular Study was approved by the local research ethics committee (OREC A: 05/Q1604/70). Written informed consent/assent was obtained from all participants.

Data sharing: No additional data available.

Transparency: The authors affirm that the manuscript is an honest, accurate, and transparent account of the study being reported; that no important aspects of the study have been omitted; and that any discrepancies from the study as planned (and, if relevant, registered) have been explained.
