# Supplementary material for: Biases in detection of apparent “weekend effect” on outcome with administrative coding data: population based study of stroke
Source: BMJ. 2016 May 16;353:i2648. doi: 10.1136/bmj.i2648 (PMC4868367; doi:10.1136/bmj.i2648)
Supplement: Supplementary file 1 — Appendix 1: Supplementary tables A-F [file lili033127.ww1_default.pdf]

Appendix 1: Supplementary tables [posted as supplied by author]

Table A Differences in inaccurate coding reasons in admissions during weekdays vs. weekend restricted to episodes coded as I60 to I64

	**Weekdays (%)**	**Weekend (%)**	**p**	**Total (%)**
**Correctly identified episodes by coding**	(n=750)	(n=278)	0.44	(n=1028)
Incident stroke	557(74.3)	213 (76.6)		770 (74.9)
Recurrent stroke	193 (15.7)	65 (23.4)		258 (25.1)
**Incorrectly identified episodes by coding**	(n=317)	(n=77)	0.02	(n=394)
Cancelled admission	2 (0.6)	0 (0)	0.49	2 (0.5)
Investigation or procedure only†	24 (7.6)	1 (1.3)	0.04	25 (6.3)
Other diagnosis‡	79 (24.9)	20 (26.0)	0.85	99 (25.1)
Rehabilitation after stroke	55 (17.4)	8 (10.4)	0.14	63 (16.0)
Medical problem post stroke discharge	19 (6.0)	4 (5.2)	0.79	23 (5.8)
Subdural/extradural haemorrhage	47 (14.8)	8 (10.4)	0.31	55 (14.0)
Transferred from other hospital	27 (8.5)	5 (6.5)	0.56	32 (8.1)
Admission date wrong	40 (12.6)	24 (31.2)	<0.0001	64 (16.2)
Inpatient event after elective admission	4 (1.3)	1 (1.3)		
Inpatient event after emergency admission for other disease	22 (6.9)	15 (19.5)		
Admission date wrong	14 (4.4)	8 (10.4)		
General practitioner information wrong	15 (4.7)	4 (5.2)	0.87	19 (4.8)
Unknown	9 (2.8)	3 (3.9)	0.63	12 (3.0)

† including carotid endarterectomy, cerebral angiography, endovascular treatment for aneurysm, neurosurgery, brain imaging
‡ including transient ischaemic attack, amaurosis fugax, trauma only, dementia, brain tumour, small vessel disease, vasculitis, seizure, headache

Table B. Differences in inaccurate coding reasons in admissions during weekdays vs. weekend restricted to episodes coded as I60, I61, I62.9, I63 and I64

	Weekdays (%)	Weekend (%)	p	Total (%)
**Correctly identified episodes by coding**	(n=748)	(n=277)	0.47	(n=1025)
Incident stroke	556 (74.3)	212 (76.5)		768 (74.9)
Recurrent stroke	192 (25.7)	65 (23.5)		257 (25.1)
**Incorrectly identified episodes by coding**	(n=266)	(n=69)	0.08	(n=335)
Cancelled admission	2 (0.8)	0 (0)	0.47	2 (0.6)
Investigation or procedure only†	21 (7.9)	1 (1.4)	0.05	22 (6.6)
Other diagnosis‡	75 (78.2)	20 (29.0)	0.9	95 (28.4)
Rehabilitation after stroke	55 (20.7)	8 (11.6)	0.09	63 (18.8)
Medical problem post stroke discharge	19 (7.1)	4 (5.8)	0.69	23 (6.9)
Subdural/extradural haemorrhage	3 (1.1)	1 (1.4)	0.83	4 (1.2)
Transferred from other hospital	27 (10.2)	5 (7.2)	0.47	32 (9.6)
Admission date wrong	40 (15.0)	23 (33.3)	0.001	63 (18.8)
Inpatient event after elective admission	4 (1.5)	1 (1.4)		
Inpatient event after emergency admission for other disease	22 (8.3)	15 (21.7)		
Admission date wrong	14 (5.3)	7 (10.1)		
General practitioner information wrong	15 (5.6)	4 (5.8)	0.96	19 (5.7)
Unknown	9 (3.4)	3 (4.3)	0.7	12 (3.6)

† including carotid endarterectomy, cerebral angiography, endovascular treatment for aneurysm, neurosurgery, brain imaging
‡ including transient ischaemic attack, amaurosis fugax, trauma only, dementia, brain tumour, small vessel disease, vasculitis, seizure, headache

Table C. Differences in 30-day case-fatality, frequency and reasons for inaccurate coding of stroke admissions during weekdays vs. weekend only including emergency cases (defined by admission method) by coding

	Total	Outcome:	Admissions:		
		Death at 30d (%)	Weekdays (%)	Weekend (%)	p
Total	(n=1327)		(n=976)	(n=351)	
**Correctly identified episodes by coding**	**1042**	**231 (22.2)†**	**761 (78.0)**	**281 80.1)**	**0.41**
**Cases of low expected case-fatality**	**238**	**35 (14.7)**	**190 (19.5)**	**48 (13.7)**	**0.015**
Elective admissions miscoded as emergency	40	7 (17.5)	33 (3.4)	7 (2.0)	0.19
Non-stroke diagnoses	198	28 (14.1)	157 (16.1)	41 (11.7)	0.047
Medical problem post stroke discharge	12	6 (50.0)	10 (1.0)	2 (0.6)	
Subdural/extradural haemorrhage	48	10 (20.8)	40 (4.1)	8 (2.3)	
Other diagnosis‡	138	12 (8.7)	107 (11.0)	31 (9.0)	
**Admission date wrong**	47	18 (38.3)	25 (2.6)	22 (6.3)	0.001
Inpatient event during admission for other disease	37	17 (45.9)	22 (2.3)	15 (4.4)	
Admission date wrong	10	1 (10.0)	3 (0.3)	7 (2.0)	

† p for difference between the correctly identified episodes vs. cases of low expected case-fatality: p=0.01
‡ including transient ischaemic attack, amaurosis fugax, trauma only, dementia, brain tumour, small vessel disease, vasculitis, seizure, headache

Table D. Accuracy of "admission method" in coding data for acute aortic events in OXVASC

	**OXVASC adjudicated (% of total)**								
**Coding recorded**	**Overall**			**Weekday**			**Weekend**		
	Elective	Emergency	Total	Elective	Emergency	Total	Elective	Emergency	Total
Elective	76 (48.4)	4 (2.5)	80	66 (52.4)	4 (3.2)	70	10 (32.3)	0 (0)	10
Emergency	9 (5.7)	68 (43.3)	77	8 (6.3)	48 (38.0)	56	1 (3.2)	20 (64.5)	21
Total	85 (54.1)	72 (45.9)	157	74 (58.7)	52 (41.3)	126	11 (35.5)	20 (64.5)	31

Table E Frequency and reasons of inaccurate coding of stroke admissions during weekdays vs. weekend stratified by OXVASC study period (30 month period)

	Overall		2002-2004		2004-2007		2007-2009		2009-2012		2012-2014	
	**Weekday†**	**Weekend**	**Weekday**	**Weekend**	**Weekday**	**Weekend**	**Weekday**	**Weekend**	**Weekday**	**Weekend**	**Weekday**	**Weekend**
**Total**	**(n=1308)**	**(n=385)**	**(n=276)**	**(n=66)**	**(n=251)**	**(n=76)**	**(n=245)**	**(n=72)**	**(n=271)**	**(n=86)**	**(n=265)**	**(n=85)**
**Correctly identified episodes by coding (true positive)‡**												
	772 (59.0)	283 (73.5)	147 (53.3)	43 (65.2)	142 (56.6)	52 (68.4)	151 (61.6)	59 (81.9)	158 (58.3)	64 (74.4)	174 (65.7)	65 (76.5)
**Incorrectly identified episodes by coding (false positive)**												
	(n=536)	(n=102)	(n=129)	(n=23)	(n=109)	(n=24)	(n=94)	(n=13)	(n=113)	(n=22)	(n=91)	(n=20)
Cancelled admission	15 (2.8)	0 (0)	0 (0)	0 (0)	0 (0)	0 (0)	3 (3.2)	0 (0)	9 (8.0)	0 (0)	3 (3.3)	0 (0)
Elective admission	267 (49.8)	26 (25.5)	60 (46.5)	7 (30.4)	49 (45.0)	6 (25.0)	51 (54.3)	3 (23.1)	60 (53.1)	6 (27.3)	47 (51.6)	4 (20.0)
Non-stroke diagnosis	183 (34.1)	43 (42.2)	41 (31.8)	7 (30.4)	46 (42.2)	14 (58.3)	30 (31.9)	7 (53.8)	32 (28.3)	5 (22.7)	34 (37.4)	10 (50.0)
Admission date wrong	46 (8.6)	26 (25.5)	16 (12.4)	6 (26.1)	9 (8.3)	3 (12.5)	8 (8.5)	2 (15.4)	9 (8.0)	9 (40.9)	4 (4.4)	6 (30.0)
GP wrong	16 (3.0)	4 (3.9)	5 (3.9)	0 (0)	3 (2.8)	1 (4.2)	2 (2.1)	1 (7.7)	3 (2.7)	2 (9.1)	3 (3.3)	0 (0)
Unknown	9 (1.7)	3 (2.9)	7 (5.4)	3 (13.0)	2 (1.8)	0 (0)	0 (0)	0 (0)	0 (0)	0 (0)	0 (0)	0 (0)

All numbers indicates number (%) unless otherwise stated.
†There is a trend of improved coding accuracy over the time both for weekday and weekend admissions (weekday - p_{trend}=0.005; weekend - p_{trend}=0.09).
‡The more false negative cases at weekday vs. weekend admissions did not change over time (p_{trend}=0.74).

Table F. Case-fatality at 30 days in weekend vs. weekdat admissions for all incident strokes that were admitted to the hospital in OXVASC stratified by study periods

Study Period	Weekend (Death/Total)	Weekday (Death/Total)	OR (95%CI)	P*	Adjusted OR† (95%CI)	p
2002-2006	21/96 (21.9)	69/261 (26.4)	0.78 (0.45-1.36)	0.38	0.79 (0.42-1.48)	0.47
2006-2010	21/111 (18.9)	69/290 (23.8)	0.75 (0.43-1.29)	0.30	0.64 (0.35-1.17)	0.15
2010-2014	22/121 (18.2)	57/288 (19.8)	0.90 (0.52-1.55)	0.71	0.90 (0.48-1.67)	0.73

*There was also no significant interaction between the study period and 'weekend effect'- p=0.71.

† adjusted for age, sex, stroke severity and baseline disability
