# Supplementary material for: Biases in detection of apparent “weekend effect” on outcome with administrative coding data: population based study of stroke
Source: BMJ. 2016 May 16;353:i2648. doi: 10.1136/bmj.i2648 (PMC4868367; doi:10.1136/bmj.i2648)

Appendix 2: Supplementary figures [posted as supplied by author]

Fig A. Modified rankin scale (mRS) at 30 days in patients admitted during weekend vs. weekdays in OXVASC excluding death at emergency department and inpatient events

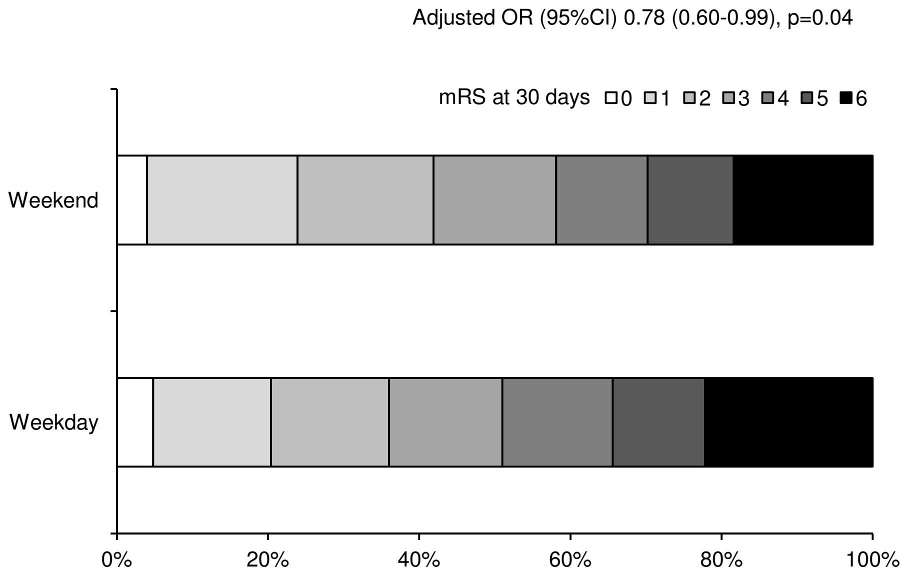

Fig B. Stroke severity (NIHSS scale) distribution of patients admitted during weekend vs. weekdays in OXVASC excluding death at emergency department and inpatient events

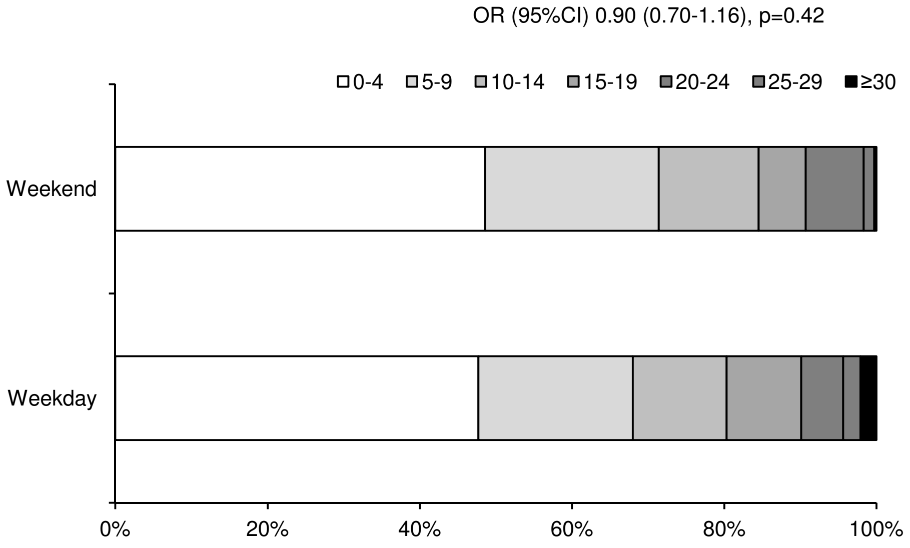

Supplement: Supplementary file 2 — Appendix 2: Supplementary figures A-B [file lili033127.ww2_default.pdf]
