# Supplementary material for: Biases in detection of apparent “weekend effect” on outcome with administrative coding data: population based study of stroke
Source: BMJ. 2016 May 16;353:i2648. doi: 10.1136/bmj.i2648 (PMC4868367; doi:10.1136/bmj.i2648)
Supplement: Supplementary file 3 — Appendix 3: Details of studies included in scoping review [file lili033127.ww3_default.pdf]

Appendix 3: Details of previous studies that studied if there is any 'weekend effect' in stroke patients [posted as supplied by author]

Year	Study period	Country	Data source	Stroke subtype	ICD codes used	Potentially inaccurate codes	If admission method available	Double-counting	Severity included	Comorbidity included	Outcome measure	'Weekend effect'
2001	1988-1997[e1]	Canada	Administrative database	ICH	431	432.9/unspecified intracranial bleed not included	Elective admissions, urgent referrals and elective transfers excluded	NR	NR	CCI	Death	No
2004	1998[e2]	US	Administrative database	Stroke	430, 431, 432, 434, 436	433 not included; 432 included SDH/EDH, 436 included seizure	Separate analyses for all admissions, all unscheduled admissions and all unscheduled admissions from ED	NR	NR	CCI	Death	No
2005	2000-2001[e3]	Japan	Prospective registry	IS/ICH	NR	-	-	NR	NIHSS	RF and premorbid disability	Death and mRS	Yes
2007	2003-2004[e4]	Canada	Administrative database	IS	I63, I64 OR 433.01, 433.11, 433.21, 433.31, 433.81, 433.91, 434.01, 434.11, 434.91	No	Not reported	NR	NR	CCI	Death	Yes
2007	1968-2005[e5]	Sweden	Administrative database	IS	NR	-	Not reported	Only first ischemic stroke	NR	NR	Death	Yes
2008	1988-2003[e6]	Japan	Prospective registry	Stroke	NR	-	-	Only first-ever stroke	NR	NR	Death	Yes
2009	2003-2006[e7]	Germany	Prospective registry	IS	I63	I64 not included	-	NR	Motor deficit, consciousness/ cardioembolic	RF and premorbid mRS	Death and mRS	No
2009	2005[e8]	Taiwan	Administrative database	IS	433.01, 433.11, 433.21, 433.31, 433.81, 433.91, 434.01, 434.11, 434.91	No	Not reported	NR	NR	CCI	Death	Yes
2009	2004-2007[e9]	US	Retrospective hospital-based study with two centres	IS/TIA/ ICH	NR	-	-	NR	NIHSS	NR	Death and mRS	No
2009	2003-2007[e10]	US	Prospective registry	IS/ICH/ SAH	430, 431, 433, 434, 436	Did not specify 433/434; 436 included seizure ; 432.9/unspecified intracranial bleed not included	-	NR	NR	RF	Death	Yes
2009	2004[e11]	US	Administrative database	ICH	431	432.9/unspecified intracranial bleed not included	Not reported	NR	NR	RF	Death	Yes
2009	2004[e12]	US	Administrative database	SAH	430	No	Not reported	NR	NR	RF	Death	No
2010	2002-2007[e13]	US	Administrative database	IS	433.01, 433.11, 433.21, 433.31, 433.81, 433.91, 434.01, 434.11, 434.91, 437.1	437.1/acute cerebrovascular insufficiency or chronic cerebral ischemia	Only admissions through ED included	NR	NR	RF	Death	No
2010	1998-2006[e14]	US	Administrative database	IS	433.01, 433.11, 433.21, 433.31, 433.81, 433.91, 434.01, 434.11, 434.91	No	Not reported	NR	NR	NR	Death	No
2010	2003-2008[e15]	Canada	Prospective registry	Stroke	NR	-	-	Only first stroke	CNS	CCI	Death	Yes
2010	1988-2003[e16]	Japan	Prospective registry	SAH	NR	-	-	Only first-ever SAH	JCS	RF	Death	No
2011	2008-2009[e17]	China	Retrospective single centre	ICH	NR	-	-	NR	ICS	NR	Death and mRS	No
2011	2006-2009[e18]	China	Retrospective single centre	SAH	NR	-	-	NR	NR	CCI	Death	No
2011	2000-2004[e19]	Holland	Administrative database	IS	433, 434, 436	Did not specify 433/434; 436 included seizure	Included also non-emergency cases but adjusted for admission method	NR	NR	CCI	Death	Yes
2011	1987-2004[e20]	US	Prospective cohort	Stroke	NR (physician-validated)	-	-	NR	Symptoms used as a proxy	CCI	Death	No

Year | Study period | Country | Data source | Stroke subtype | ICD codes used | Potentially inaccurate codes | If admission method available | Double-counting | Severity included | Comorbidity included | Outcome measure | 'Weekend effect'

2011	1996-2007[e21]	US	Administrative database	IS	433.01, 433.11, 433.21, 433.31, 433.81, 433.91, 434.01, 434.11, 434.91	No	Not reported	Only the first discharge record	NR	RF	Death	Yes
2012	2004-2005[e22]	Korea	Prospective registry	IS	NR	-	-	NR	NIHSS	RF and premorbid mRS	Death and mRS	No
2012	2004-2005[e23]	Poland	Prospective registry	IS	NR	-	-	NR	Consciousness/ cardioembolic	RF and premorbid mRS	mRS	Yes
2012	2008-2009[e24]	Spain	Prospective single centre	ICH/IS	NR	-	-	NR	NIHSS	RF	mRS	No
2012	2009-2010[e25]	UK	Administrative database	Acute CVD	G436, I60, I61, I62, I63, I66	I62 included SDH/EDH, I66/occlusion without infarct, I64 not included	Stratified by emergency vs. elective admissions but not reported for stroke	NR	NR	CCI, admission route	Death	Yes
2012	2009-2010[e26]	UK	Administrative database	Stroke	I60-I64	I62 included SDH/EDH	Admission method known but not used in analyses	NR	NR	CCI	Death	Yes
2012	2002-2009[e27]	US	Prospective registry	IS	NR	-	-	NR	NIHSS	NR	Death and mRS	No
2013	2006-2008[e28]	Brazil	Prospective single centre	IS/ICH	NR	-	-	Only first-time stroke	NR	RF	Death	No
2013	1985-2010[e29]	France	Population-based cohort	Stroke/ TIA	NR (multiple sources)	-	-	Only first-in-study events	NIHSS	RF	Death	No
2014	2000-2007[e30]	Australia	Administrative database	Stroke	ICD 10/DRG B70	Included TGA, SDH and occlusion of artery without infarct, and other CVD	Only included admissions through ED	When patient readmitted within 1week of discharge, only the last episode of care is taken	NR	CCI	Death	Yes
2014	2011-2012[e31]	England	Prospective registry	IS/ICH	NR	-	-	NR	Consciousness	Premorbid functional status	Death	No
2014	2010-2011[e32]	Japan	Administrative database	Stroke	I60.0-I60.9, I61.0-I61.9, I62.0, I62.1, I62.9, I63.0-I63.9	SDH/EDH included; I64 not included	Not reported	NR	JCS	RF	mRS	No
2014	2009-2013[e33]	US	Retrospective single centre	ICH	NR	-	-	NR	Yes/no details	NR	mRS	No
2015	2010[e34]	Denmark	Administrative database	Stroke	I60, I61, I63, I64	I62.9 unspecified intracranial haemorrhage not included	Subgroup analyses including only admissions from ED for all but not reported for stroke	Only first acute admission	NR	CCI	Death	Yes
2015	-[e35]	Spain	Retrospective single centre	Stroke	NR	-	-	NR	NIHSS	NR	mRS	No
2015	2004-2011[e36]	UK	Administrative database	IS/ICH/ unknown stroke	I61-I64	I62 included SDH/EDH	Not reported	Only the first admission	NR	RF	Death	Yes
2015	2013-2014[e37]	UK	Administrative database	Cardiovas cular disease	ICD 10 but no detailed codes reported	-	Adjusted for admission urgency	NR	NR	CCI/predicted mortality risk	Death	Yes
2015	2004-2012[e38]	England &Wales	Administrative database	Stroke/ SAH	I60, I61-64	I62 included SDH/EDH	Not mentioned how emergency admissions were defined	NR	NR	NR	Death	Yes
2015	2010-2011[e39]	Japan	Administrative database	IS	I63	I64 not included	Not reported	NR	NR	RF and premorbid mRS	Death and mRS	No
2015	2002-2012[e40]	Japan	Prospective single centre	IS	NR	-	-	NR	NIHSS	RF and premorbid mRS	mRS	No
2016	2005-2013[e41]	Scotland	Administrative database	IS/ICH	NR	-	Not reported	NR	NR	RF	Death	Yes
2016	2002-2013[e42]	Korea	Administrative database	IS	I63, G45	TIA included; I64 not included	Not reported	NR	NR	CCI	Death	Yes

IS=ischaemic stroke, ICH=intracerebral haemorrhage, SAH=subarachnoid haemorrhage, EDH=extradural haemorrhage, SDH=subdural haemorrhage, TIA=transient ischaemic attack, TGA=transient global amnesia, CVD=cerebrovascular disease, ED=Emergency Department, NIHSS=The National Institutes of Health Stroke Scale, JCS=Japan Coma Scale, CNS=Canadian Neurological Scale, ICS=Intracerebral haemorrhage Score, CCI=Charlson Comorbidity Index, RF=risk factors, mRS=modified Rankin Scale, NR=not reported.

e1. Bell CM, Redelmeier DA. Mortality among patients admitted to hospitals on weekends as compared with weekdays. *N Engl J Med* 2001; 345: 663-8.

e2. Cram P, Hillis SL, Barnett M, Rosenthal GE. Effects of weekend admission and hospital teaching status on in-hospital mortality. *Am J Med* 2004; 117: 151-7.

e3. Hasegawa Y, Yoneda Y, Okuda S, et al. The effect of weekends and holidays on stroke outcome in acute stroke units. *Cerebrovasc Dis* 2005; 20: 325-31.

e4. Saposnik G, Baibergenova A, Bayer N, Hachinski V. Weekends: a dangerous time for having a stroke? *Stroke* 2007; 38: 1211-5.

e5. Janszky I, Ahnve S, Ljung R. Weekend versus weekday admission and stroke outcome in Sweden from 1968 to 2005. *Stroke* 2007; 38: e94.

e6. Turin TC, Kita Y, Rumana N, et al. Case fatality of stroke and day of the week: is the weekend effect an artifact? Takashima stroke registry, Japan (1988-2003). *Cerebrovasc Dis* 2008; 26: 606-11.

e7. Jauss M, Oertel W, Allendoerfer J, Misselwitz B, Hamer H. Bias in request for medical care and impact on outcome during office and non-office hours in stroke patients. *Eur J Neurol* 2009; 16: 1165-7.

e8. Tung YC, Chang GM, Chen YH. Associations of physician volume and weekend admissions with ischemic stroke outcome in Taiwan: a nationwide population-based study. *Med Care* 2009; 47: 1018-25.

e9. Albright KC, Raman R, Ernstrom K, et al. Can comprehensive stroke centers erase the 'weekend effect'? *Cerebrovasc Dis* 2009; 27: 107-13.

e10. Reeves MJ, Smith E, Fonarow G, et al. Off-hour admission and in-hospital stroke case fatality in the get with the guidelines-stroke program. *Stroke* 2009; 40: 569-76.

e11. Crowley RW, Yeoh HK, Stukenborg GJ, Medel R, Kassell NF, Dumont AS. Influence of weekend hospital admission on short-term mortality after intracerebral hemorrhage. *Stroke* 2009; 40: 2387-92.

e12. Crowley RW, Yeoh HK, Stukenborg GJ, Ionescu AA, Kassell NF, Dumont AS. Influence of weekend versus weekday hospital admission on mortality following subarachnoid hemorrhage. Clinical article. *J Neurosurg* 2009; 111: 60-6.

e13. Hoh BL, Chi YY, Waters MF, Mocco J, Barker FG, 2nd. Effect of weekend compared with weekday stroke admission on thrombolytic use, in-hospital mortality, discharge disposition, hospital charges, and length of stay in the Nationwide Inpatient Sample Database, 2002 to 2007. *Stroke* 2010; 41: 2323-8.

e14. Kazley AS, Hillman DG, Johnston KC, Simpson KN. Hospital care for patients experiencing weekend vs weekday stroke: a comparison of quality and aggressiveness of care. *Arch Neurol* 2010; 67: 39-44.

e15. Fang JM, Saposnik G, Silver FL, Kapral MK, Network RCS. Association between weekend hospital presentation and stroke fatality. *Neurology* 2010; 75: 1589-96.

e16. Turin TC, Kita Y, Rumana N, et al. Circaseptan variation in case-fatality rate for patients with acute subarachnoid hemorrhage (Takashima Stroke Registry 1988-2003). *J Clin Neurosci* 2010; 17: 869-73.

e17. Jiang F, Zhang JH, Qin X. "Weekend effects" in patients with intracerebral hemorrhage. *Acta Neurochir Suppl* 2011; 111: 333-6.

e18. Zhang G, Zhang JH, Qin X. Effect of weekend admission on in-hospital mortality after subarachnoid hemorrhage in Chongqing China. *Acta Neurochir Suppl* 2011; 110: 229-32.

e19. Ogbu UC, Westert GP, Slobbe LC, Stronks K, Arah OA. A multifaceted look at time of admission and its impact on case-fatality among a cohort of ischaemic stroke patients. *J Neurol Neurosurg Psychiatry* 2011; 82: 8-13.

e20. O'Brien EC, Rose KM, Shahar E, Rosamond WD. Stroke Mortality, Clinical Presentation and Day of Arrival: The Atherosclerosis Risk in Communities (ARIC) Study. *Stroke Res Treat* 2011; 2011: 383012.doi: 10.4061/2011/383012.

e21. McKinney JS, Deng Y, Kasner SE, Kostis JB, Myocardial Infarction Data Acquisition System Study G. Comprehensive stroke centers overcome the weekend versus weekday gap in stroke treatment and mortality. *Stroke* 2011; 42: 2403-9.
e22. Kim SC, Hong KS, Hwang SI, et al. Weekend admission in patients with acute ischemic stroke is not associated with poor functional outcome than weekday admission. *J Clin Neurol* 2012; 8: 265-70.
e23. Niewada M, Jezierska-Ostapczuk A, Skowronska M, Sarzynska-Dlugosz I, Czlonkowska A. Weekend versus weekday admissions in Polish stroke centres -- could admission day affect prognosis in Polish ischaemic stroke patients? *Neurologia i neurochirurgia polska* 2012; 46: 15-21.
e24. Martinez-Martinez MM, Fernandez-Travieso J, Fuentes B, et al. Off-hour effects on stroke care and outcome in stroke centres. *Eur J Neurol* 2012; 19: 1140-5.
e25. Freemantle N, Richardson M, Wood J, et al. Weekend hospitalization and additional risk of death: an analysis of inpatient data. *Journal of the Royal Society of Medicine* 2012; 105: 74-84.
e26. Palmer WL, Bottle A, Davie C, Vincent CA, Aylin P. Dying for the weekend: a retrospective cohort study on the association between day of hospital presentation and the quality and safety of stroke care. *Arch Neurol* 2012; 69: 1296-302.
e27. Albright KC, Savitz SI, Raman R, et al. Comprehensive stroke centers and the 'weekend effect': the SPOTRIAS experience. *Cerebrovasc Dis* 2012; 34: 424-9.
e28. Barros JB, Goulart AC, Alencar AP, Lotufo PA, Bensenor IM. The influence of the day of the week of hospital admission on the prognosis of stroke patients. *Cadernos de saude publica* 2013; 29: 769-77.
e29. Bejot Y, Aboa-Eboule C, Jacquin A, et al. Stroke care organization overcomes the deleterious 'weekend effect' on 1-month stroke mortality: a population-based study. *Eur J Neurol* 2013; 20: 1177-83.
e30. Concha OP, Gallego B, Hillman K, Delaney GP, Coiera E. Do variations in hospital mortality patterns after weekend admission reflect reduced quality of care or different patient cohorts? A population-based study. *BMJ Qual Saf* 2014; 23: 215-22.
e31. Bray BD, Ayis S, Campbell J, et al. Associations between stroke mortality and weekend working by stroke specialist physicians and registered nurses: prospective multicentre cohort study. *PLoS Med* 2014; 11: e1001705. doi: 10.1371/journal.pmed.1001705.
e32. Kamitani S, Nishimura K, Nakamura F, et al. Consciousness level and off-hour admission affect discharge outcome of acute stroke patients: a J-ASPECT study. *J Am Heart Assoc* 2014; 3: e001059. doi: 10.1161/JAHA.114.001059.
e33. McDowell MM, Kellner CP, Sussman ES, et al. The role of admission timing in the outcome of intracerebral hemorrhage patients at a specialized stroke center. *Neurol Res* 2014; 36: 95-101.
e34. Vest-Hansen B, Riis AH, Sorensen HT, Christiansen CF. Out-of-hours and weekend admissions to Danish medical departments: admission rates and 30-day mortality for 20 common medical conditions. *BMJ open* 2015; 5: e006731. doi: 10.1136/bmjopen-2014/006731
e35. Romero Sevilla R, Portilla Cuenca JC, Lopez Espuela F, et al. A stroke care management system prevents outcome differences related to time of stroke unit admission. *Neurologia* 2015. doi: 10.1016/j.nrl.2015.07.011.
e36. Roberts SE, Thorne K, Akbari A, Samuel DG, Williams JG. Mortality following Stroke, the Weekend Effect and Related Factors: Record Linkage Study. *PLoS One* 2015; 10: e0131836. doi: 10.1371/journal.pone.0131836
e37. Freemantle N, Ray D, McNulty D, et al. Increased mortality associated with weekend hospital admission: a case for expanded seven day services? *BMJ* 2015; 351: h4596. doi: 10.1136/bmj.h4596
e38. Roberts SE, Thorne K, Akbari A, Samuel DG, Williams JG. Weekend emergency admissions and mortality in England and Wales. *Lancet* 2015; 385: 1829.

e39. Inoue T, Fushimi K. Weekend versus Weekday Admission and In-Hospital Mortality from Ischemic Stroke in Japan. *J Stroke Cerebrovasc Dis* 2015; 24: 2787-92.
e40. Nakajima M, Inatomi Y, Yonehara T, Watanabe M, Ando Y. Outcome in patients admitted outside regular hospital working hours: does time until regular working hours matter? *Int J Stroke* 2015; 10: 79-84.
e41. Turner M, Barber M, Dodds H, Dennis M, Langhorne P, Macleod MJ. Stroke patients admitted within normal working hours are more likely to achieve process standards and to have better outcomes. *J Neurol Neurosurg Psychiatry* 2016; 87: 138-43.
e42. Cho KH, Park EC, Nam CM, Choi Y, Shin J, Lee SG. Effect of Weekend Admission on In-Hospital Mortality in Patients with Ischemic Stroke: An Analysis of Korean Nationwide Claims Data from 2002 to 2013. *J Stroke Cerebrovasc Dis* 2016; 25: 419-27.
